# Identifying affective personality profiles: A latent profile analysis of the Affective Neuroscience Personality Scales

**DOI:** 10.1038/s41598-017-04738-x

**Published:** 2017-07-03

**Authors:** Massimiliano Orri, Jean-Baptiste Pingault, Alexandra Rouquette, Christophe Lalanne, Bruno Falissard, Catherine Herba, Sylvana M. Côté, Sylvie Berthoz

**Affiliations:** 1 CESP, INSERM, Univ. Paris-Sud, UVSQ, Université Paris-Saclay, Villejuif, France; 2 Bordeaux Population Health Research Centre, INSERM U1219, University of Bordeaux, Bordeaux, France; 3 Research unit on children’s psychosocial maladjustment, Centre de Recherche du CHU Sainte-Justine, University of Montreal, Montreal, Canada; 4 Division of Psychology and Language Sciences, University College London, London, UK; 5 AP-HP, Hôtel-Dieu Hospital, Biostatistics and Epidemiology Department, Paris, France; 6 AP-HP, Saint-Louis Hospital, Department of Clinical Research, Paris, France; 7 Department of Psychology, Université du Québec à Montréal, Montreal, Canada; 8 Tomsk State University, Russian Federation, Tomsk, Russia; 9 Institute Mutualiste Montsouris, Department of Adolescent and Young Adult Psychiatry, Paris, France

## Abstract

Based on evolutionary theory, a recent model in affective neuroscience delineated six emotional brain systems at the core of human personality: SEEKING, CARING, PLAYFULNESS, FEAR, ANGER, SADNESS. The Affective Neuroscience Personality Scales (ANPS) assess their functioning. Using a person-centred approach of the ANPS, this study: (i) examined the existence of latent personality profiles, (ii) studied their gender invariance, (iii) assessed their longitudinal (4 years) stability, and (iv) explored how they relate to several intrapersonal, interpersonal, and emotion regulation skills. Latent Profile Analysis in 2 samples (Canadian, longitudinal, N = 520; French, cross-sectional, N = 830) found that, qualitatively, 3 profiles characterized both populations and genders, with one distinction for the second profile where the French women endorsed slightly higher and lower scores for, respectively, the negative and positive emotions. Whilst not being quantitatively similar across genders, the personality profiles remained consistent across time in the longitudinal sample. Associations between profiles and intrapersonal (e.g. depression), interpersonal (e.g. empathy), and emotion regulation skills measures (e.g. emotional intelligence) offered concurrent validity evidence. This person centred approach to ANPS offers a holistic and parsimonious way to study affective personality dimensions. It opens promising avenues for future studies on the predictive value of ANPS profiles, and for personality-targeted interventions.

## Introduction

## Personality from the perspective of affective neurosciences

Emotions and emotional regulation processes are at the basis of human personality, as well as of many psychological and psychiatric disorders^1–3^. Differences in the expression and regulation of emotions account for a large extent of the individual differences in personality^4^.

Relying on neurobiology, ethology, and evolutionary findings, Panksepp and collaborators examined brain systems at the foundation of our emotions^5, 6^. Panksepp’s approach was operationalized by Davis *et al*. with a psychometric self-report instrument, the Affective Neuroscience Personality Scales (ANPS)^7^. The ANPS measure three positive and three negative emotional systems at the core of human emotional processes^8^ (capitalizations reflect a specialized scientific terminology): (1) SEEKING/interest, described as being curious, exploring, striving for solutions to problems, positively anticipating new experiences; (2) CARING/nurturance, described as being drawn to young children and pets, feeling soft-hearted toward animals and people in need, feeling empathy; (3) PLAYFULNESS/joy, described as having fun, playing games with physical contacts, humour, and laughter; (4) FEAR/anxiety, described as feeling tense, worrying, struggling with decisions, ruminating; (5) ANGER/rage, described as feeling hot-headed, being easily irritated and frustrated, experiencing frustration leading to anger, expressing anger verbally or physically; (6) SADNESS/panic and separation distress, described as feeling lonely, crying frequently, thinking about loved ones and past relationships, and feeling distress. These systems are conceptualized as *emotional endophenotypes*, i.e. emotional markers of underlying neuropsychological activities that lie between genes and behaviours^9^. These primary affective networks mould the development of higher-order mental skills and frame the individual’s subjective feelings, behaviours and relationships^9–11^.

With this respect, the underlying hypothesis of the development of the ANPS differs from that of personality scales relying on a lexical approach, such as the Five Factor Model (FFM). The lexical approach posits that “most of the socially relevant and salient personality characteristics have become encoded in the natural language” (p. 103)^12^. Thus the FFM measures were not elaborated with the aim of reflecting underlying biological processes^13–15^.

There is an increasing number of neurobiological studies using the ANPS. They offer validity evidence based on the observed relations between the ANPS scores and genetic markers (e.g., FEAR and SADNESS with the serotonin transporter polymorphism and the oxytocin receptor gene markers; ANGER with dopaminergic polymorphism) or cerebral morphological and functional substrates (e.g., ANGER or FEAR scores and the volume of the amygdala; SADNESS and the level of activity or of functional connectivity of fronto-limbic regions)^13, 14, 16–20^.

## Person-centred approach to personality

Until recently, studies using the ANPS have focused either on associations with a single dimension (e.g. the association between SEEKING and depression)^21^, or have explored the role of each dimension separately (e.g., using simple regressions)^22^, or simultaneously (e.g., using multiple regressions or Principal Components Analysis)^23, 24^. All these methods have in common a *variable-centred* approach to the ANPS dimensions, which does not account for the way these dimensions relate within individuals. Indeed, a *variable-centred* approach assumes that the whole population is homogenous and the results are thus an estimate of the relationships averaged over the whole population. In contrast, *person-centred* approaches “describe similarity and differences among individuals with respect to how variables relate to each other” (page 552)^25^. For instance, a variable-centred approach analysis could reveal that on average the ANPS positive emotions are higher than the negative ones in a given population. In contrast, a person-centred approach in this same population may uncover two qualitatively different (and more homogenous) subgroups: the first one characterized by people having high positive emotions and very low negative emotions, and the second one characterized by people having high scores on both positive and negative emotions. These two qualitative different subgroups (or personality typologies) are not revealed by the variable-centred approaches because such approaches assume the whole population is homogenous. Therefore, the results are an average estimate of the relationships observed in the whole sample. Rather, in person-centred approaches the population is assumed to be heterogeneous and, for instance, in the context of the ANPS, these person-centred approaches may uncover a number of subpopulations (i.e. latent classes, not directly observed) in which people present similar patterns of emotions. The distinction of these subpopulations may be useful to study the relationships between variables that may differ quantitatively or qualitatively between these subpopulations^26^ and also to better study the balance between positive and negative emotional systems, as proposed by Panksepp and collaborators^9, 10^. Another advantage of person-centred approaches is that the typologies (i.e., subpopulations) are empirically derived, and the existence of the subpopulation is statistically tested. For instance, researchers could create typologies by classifying people obtaining the upper 20% scores on the variables of interest in one profile, those obtaining the lower 20% scores in a second profile, and those obtaining intermediate scores in a third profile. However, doing so, the researchers arbitrarily define the cut-off, and profiles would change with another cut-off. More importantly, the existence of subgroups within the population is never tested, that is no model assuming no-subpopulations is tested against a model assuming two or more subpopulations in order to verify which one fits the data best (i.e., verifying the hypothesis of population heterogeneity).

To our knowledge, no study in clinical or population-based samples has adopted a person-centred approach to the ANPS. Latent Profile Analysis (LPA), a specific case of a finite mixture model^27^, is a person-centred approach that enables to identify subgroups (i.e. profiles, or latent classes) of people according to the patterns of relationships among some measured continuous variables (i.e. *indicators* in mixture modelling terminology). The goal of LPA is to identify the fewest number of latent classes (i.e. homogenous groups of individuals) that adequately explain the unobserved heterogeneity of the relationships between indicators within a population.

Surprisingly, person-centred approaches are relatively uncommon in personality research. Specht and collaborators^28^ reported that up to 2014, only 14 studies (15 with the authors’ empirical contribution) analysed personality typologies in adolescent and adult populations, and consistently described 3 typologies (resilients, overcontrolled, and undercontrolled). In all of these studies, however, personality was assessed using questionnaires based on the Five Factor Model or relying on a lexical approach of personality^12^. This study is the first to date to use a person-centred approach with the ANPS. Since the ANPS is based on an alternative approach to personality theory, with strong biological and evolutionary underpinnings, it is relevant to investigate the typologies emerging from the study of personality as assessed by these scales.

## Aims of the present study

The present study examined profiles emerging from the study of personality using the ANPS by applying LPA to data from 2 different cohorts of French-speaking young adults (one Canadian longitudinal sample, the other French cross-sectional sample). Our specific objectives were:

(i) To describe the ANPS profiles in both men and women. Since this is the first study interested in typologies of personality assessed using the ANPS, no hypotheses were formulated concerning the number of latent profiles. However, we expected similar profiles to emerge in the 2 cohorts.
(ii) To explore differences in these profiles across genders. Measurement model invariance of the ANPS across genders has already been demonstrated^29^, which guarantees that the analyses performed in the present study, at the level of the mean and covariance structure, are meaningful. Based on previous studies indicating greater propensity for nurturing and for anxious/depressive affects among women^30–32^, we expected higher level of CARING, FEAR, and SADNESS in women.
(iii) To evaluate the stability of the profiles over time (4 years). Longitudinal measurement invariance of the ANPS has been demonstrated, as well as the good time stability of the 6 dimensions assessed in a sample of adults followed over a 4 years period^29^, providing support that the scales measure personality traits. Thus, we also expected longitudinal consistency for the ANPS profiles.
(iv) To assess the concurrent validity of the ANPS profiles by exploring the associations between ANPS profiles and several intrapersonal (depression, anxiety, anger, and positive/negative affectivity), interpersonal (cognitive and emotional dimensions of empathy), and emotion regulation constructs (emotional intelligence domains).

## Methods

### Samples

Two different samples of French-speakers were used: Canadian (Sample 1, longitudinal), and French (Sample 2, cross-sectional).

Participants in Sample 1 came from the EMIGARDE cohort, a longitudinal study of child development conducted in Montreal (Quebec, Canada) from 2003 to 2011 with 4 collection times (2004-2005-2006-2010). Families included in the EMIGARDE cohort were drawn from a larger pool (N = 809) of families with a child born between June 2003 and April 2004 and having taken part in a prenatal–perinatal study conducted in four Montreal maternity hospitals (for details, see^33^). A subgroup of fathers and mothers of these children completed the ANPS at the 2 last data collection points, in 2006 (T1, N = 520) and 2010 (T2, N = 569). The exclusion of individuals with questionnaires having more than 10% of missing items (N = 11 at T1 and N = 1 at T2) resulted in a final sample of 509 participants (222 men, and 287 women) at T1 and 568 participants (249 men, and 319 women) at T2. At T1, the mean age of the men and women was 38.4 ± 6.3 and 35.2 ± 5.0 years respectively (overall mean age: 36.5 ± 5.8 years). Most participants had an intermediate to high level of education: university degree (56.4%), high school grade (24.7%), partial college education (8.6%), and partial secondary education (7.9%); 2.4% had no secondary education. Concerning the ethnicity, the origin of most participants was Canadian (65%) or French (8%), and the remaining participants had other ethnical origins (27%; mainly British and Italian). The study received approval from Sainte-Justine Hospital research centre and McGill Institutional Review Boards. Participants gave their written informed consent at each data collection, and all procedures were carried out in accordance with the relevant guidelines and regulations.

Participants in Sample 2 came from the DETENDOEMO survey, conducted in France in 2008. The study was approved by the Ethics Committee Paris Ile-de-France VI, and all participants gave their written informed consent. All procedures were carried out in accordance with the relevant guidelines and regulations. One thousand questionnaires were administered to college students of various disciplines from different universities or higher education institutions located in the Parisian area (social work, psychology, art, biology and biotechnologies, computer science, and general engineering) during class. Of the returned questionnaires, 32 were excluded because they were incomplete or because the identity or consent form was missing. This resulted in a final sample of 830 young adults (mean age 20.6 ± 2.1), including 54.8% women (mean age 20.6 ± 2.0) and 45.2% men (mean age 20.7 ± 2.3). Except for 2 participants who did not complete high school (they were assistants that were presents at the moments of the administration), 31.2% graduated from high school, 39.2% completed at least 2 years of college, and 29.6% less than 2 years of college. Information regarding the ethnicity of these participants was not available, as the ability to collect this information is extremely restricted by the French legislation and must be duly substantiated.

The socio-demographic characteristics of the two samples are summarized in the Supplementary Table S1.

### Measures

The French translation of the ANPS (version 2.4)^34, 35^ was used in both samples. Each ANPS subscale comprised 14 items, answered on a 4-point Likert scale (*totally disagree-disagree-agree-totally agree*). The 14 items (half are reverse coded) are summed to obtain the score of the subscale. Previous validation analyses using this instrument have been conducted to ascertain its psychometric properties: good reliabilities of the ANPS (Cronbach alphas range: 0.77–0.89), gender and longitudinal measurement invariance, mean score stability over time, and external validity^29, 35, 36^.

In addition, in Sample 2, several instruments were administered (all validated in French) in order to assess (i) intrapersonal constructs, (ii) interpersonal constructs, and (iii) emotion regulation skills. The following instruments (α refers to Cronbach’s alpha in the present sample) were used:

- Measures of intrapersonal constructs:
  The *Beck Depression Inventory* short form (BDI 13 items, score ranging from 0 to 26, α = 0.79) evaluating depressive affects and cognitions. High values indicate depression^37, 38^.
  The *State and Trait Anxiety Inventory, Trait Scale* (STAI Trait, 20 items, score ranging from 20 to 80, α = 0.89). High values indicate the presence of trait anxiety^39, 40^.
  The *Multidimensional Anger Inventory* (MAI, 38 items, score ranging from 38 to 190, α = 0.86). High total scores indicate feelings of anger and associated behaviours^41, 42^.
  The *Positive and Negative Emotionality Questionnaire* (EPN-31, 31 items): elaborated according to Diener^43^ and the tripartite model of affects^44^. It comprises 31 French words corresponding to positive and negative emotions or affects. Global positive and negative emotion scores are calculated (α = 0.84 and 0.92 respectively). Positive and negative emotion scores range from 10 to 70 and from 18 to 126 respectively, with high scores corresponding to high emotions^45^.
- Measures of interpersonal constructs:
  The *Interpersonal Reactivity Index* ^46, 47^ assessing affective (Empathic Concern, IRI-EC, α = 0.34; Personal Distress, IRI-PD, α = 0.62) and cognitive (Fantasy, IRI-F, α = 0.52, Perspective Taking, IRI-PT, α = 0.36) components of empathy. Each scale ranges from 0 to 28, with high scores representing high values of the measured construct.
- Measures of emotional regulation skills:

The *Trait Meta-Mood Scale* (TMMS, 30 items)^48, 49^, assessing individuals’ beliefs about their own emotional abilities. It comprises 3 subscales: Attention towards emotions (TMMS-A, α = 0.85, range 14 to 70, i.e. the tendency to observe and think about their own emotions), Clarity of feelings (TMMS-C, range 10 to 50, α = 0.79, i.e. the understanding of one’s emotional states), and Repair (TMMS-R, range 6 to 30, α = 0.74, i.e. the ability to regulate their emotions). High scores indicate better emotional skills.

Additionally, social desirability was assessed using the short version of the Social Desirability Scale^50, 51^. The score ranges from 0 to 10, high scores represent response biased by social desirability.

### Determination and description of the latent profiles

We decided to study ANPS profiles in men and women separately because significant gender differences in the ANPS dimensions have been regularly found in the literature^7, 29, 34, 35^. LPA models with 1 to 5 latent classes were thus sequentially fitted separately for each gender group on both samples (at T1 for sample 1). The best model was chosen according to the procedure delineated by Nylund^52^, evaluating several fit indices: i) the Bayesian Information Criterion (BIC; smaller values indicate better model)^53^, ii) the Vuong-Lo-Mendel-Rubin Likelihood Ratio Test (LMR)^54^ which compares the fit of models with N or N-1 classes (a p-value < 0.05 suggesting that the additional class improves the fit of the model), and iii) the entropy, indicating the accuracy with which models classify individuals into their most likely class (range 0–1, higher values indicating better classification accuracy). Among those indices, the BIC seems to be the most reliable for continuous latent class models according to simulation studies^52^. The interpretability of the classes based on theoretical considerations, the shape of the profiles (i.e. the pattern among the six indicators), and the classes’ size (i.e. number of participants within each class), were also taken into account when deciding about the number of latent classes. Indeed, relying exclusively on fit indices can lead to misinterpretation of the empirical results.

After the selection of the best model in each sample and gender, individuals were assigned to the profile (i.e. the latent class) according to their highest posterior probability of class membership. Then, the score for each of the six dimensions in each profile was described using means and standard deviations (SD).

### Assessment of latent class invariance across gender

The aim of this analysis was to determine if the profiles found in both genders were quantitatively similar. It was performed separately on Sample 1 (T1) and Sample 2 using Multiple Groups Latent Profile Analysis (MGLPA). A preliminary step was to perform a qualitative comparison between men and women profiles, in order to establish which pairs of profiles (one among men, one among women) had to be formally compared in the invariance assessment process. Our criterion for this qualitative comparison was the overall pattern among the six ANPS emotions, and in particular the balance between positive and negative emotions, which is a key aspect of Panksepp’s theory^7–9^. Measurement invariance testing followed three steps delineated by Collins & Lanza^55^. After having determined whether the number of latent classes were the same in both groups as described above (first step), invariance of indicator means was tested using nested models (second step): an unconstrained model (M1) with freely estimated indicator means in both groups; a constrained model (M2 nested in M1) with equality constraint on indicator means across groups. If LPA models were not found to be invariant across groups, partial invariance was tested by relaxing one equality constraint at a time until partial invariance was reached. If only partial invariance was reached at the level of indicator means, the process ended, but if the hypothesis of total indicator means invariance held, the invariance of latent classes proportions across groups was tested using a third model (M3 nested in M2) with an additional equality constraint on latent classes proportions (third step).

The Likelihood ratio test was used in order to compare the nested models.

### Stability over time of the ANPS profiles

These analyses were performed on Sample 1 only as their aim was to study the longitudinal stability of the profiles that emerged from LPA at T1. We used Latent Profile Transition Analysis (LPTA, a longitudinal extension of LPA) in which transitions between latent classes from T1 to T2 are allowed for each subject of the sample. The latent transition probability matrix (which expresses the probability of a change of latent class membership over time conditional on previous class membership) was analysed to determine the extent to which latent profile membership was stable over time.

### Association with interpersonal, intrapersonal, and emotional regulation skills constructs

This analysis was performed only in Sample 2 in which interpersonal and intrapersonal, and emotional regulation skills constructs were measured. We compared how the different profiles (independent variables) may differ on these variables (dependent variables) using analysis of covariance (ANCOVA) to adjust for the Social Desirability scale’s score. Next, planned adjusted comparisons between pairs of profiles were conducted. All statistical tests were two-tailed and the Type I error rate was fixed at 5%. A Bonferroni correction was applied in order to take into account the alpha level inflation due to multiple comparisons^56^ Effect sizes (Hedge’s g) were calculated and interpreted according to Cohen (1988) as follows: <0.20 = small; 0.21–0.50 = medium; 0.51–0.80 = large; >0.80 = very large^57^.

### Software

Data management, descriptive analyses, and bivariate analyses were performed using R version 3.0^58^. LPA and LPTA analyses were performed using M*plus* version 7.4^59^ with maximum likelihood and a robust estimator (Huber-White, MLR estimator in M*plus*) in order to handle the non-normal distribution of the indicators. The MLR estimator gives correct chi-square based statistics and standard errors, thus handling potential issue of leptokurtotic and platykurtotic data. Full Information Maximum Likelihood (FIML) was used to handle missing data^60^.

## Results

### Profiles of the ANPS in the 2 samples

The fit statistics of the LPA models for men and women in each sample are reported in Table 1, with the proportions of women and men in each profile. Residual covariances were generally acceptable, indicating no meaningful violation of local independence (i.e., the indicators of the latent variable are conditionally independent of each other given the score on the latent variable). In both samples, the BIC reached a minimum in the 3-class or 4-class solution for both men and women. The 4-classes solution was generally not supported by LMR test, had lower entropy levels, small class sizes, and appeared less theoretically sound (i.e. the fourth profile did not add substantial information). Therefore, 3 profiles were selected for each sample.

**Table 1:** Fit indices for the selected LPA models in Sample 1 and Sample 2. Statistical indices of the estimated LPA models. LL = loglikelihood; k = number of parameters; BIC = Bayesian Information Criterion; LMR = p value of the Vuong-Lo-Mendell-Rubin Likelihood Ratio Test. P1-P5 = Profile 1-Profile 5.

	LL (k)	BIC	Entropy	LMR	Class size N (%)				
					P1	P2	P3	P4	P5
**Sample 1**									
**Women (N = 287)**									
1 class	−2376.619 (12)	4821.153	1	—	287 (100)	—	—	—	—
2 classes	−2298.254 (19)	4704.039	0.680	0.289	199 (69.3)	88 (30.7)	—	—	—
3 classes	−2248.481 (26)	4644.108	0.768	0.071	68 (23.7)	186 (64.8)	33 (11.5)	—	—
4 classes	−2228.292 (33)	4643.347	0.701	0.034	67 (23.3)	132 (46.0)	57 (19.9)	31 (10.8)	—
5 classes	−2212.338 (40)	4651.055	0.745	0.327	67 (23.3)	126 (43.9)	64 (22.3)	15 (5.2)	15 (5.2)
**Men (N = 222)**									
1 class	−1836.507 (12)	3737.847	1	—	222 (100)	—	—	—	—
2 classes	−1794.011 (19)	3690.674	0.627	0.184	68 (30.6)	154 (69.4)	—	—	—
3 classes	−1767.542 (26)	3675.553	0.733	0.070	60 (27.0)	142 (64.0)	20 (9.0)	—	—
4 classes	−1749.045 (33)	3676.378	0.749	0.394	77 (34.7)	113 (50.9)	26 (11.7)	6 (2.7)	—
5 classes	−1735.189 (40)	3686.484	0.711	0.355	77 (34.7)	105 (47.3)	26 (11.7)	10 (4.5)	4 (1.8)
**Sample 2**									
**Women (N = 455)**									
1 class	−3776.967 12)	7627.378	1	—	455 (100)	—	—	—	—
2 classes	−3638.339 (19)	7392.963	0.706	0.000	244 (53.6)	211 (46.4)	—	—	—
3 classes	−3591.158 (26)	7341.444	0.702	0.116	225 (49.9)	66 (14.4)	163 (36.0)	—	—
4 classes	−3553.455 (33)	7308.880	0.710	0.796	185 (40.7)	197 (43.3)	51 (11.2)	22 (4.8)	—
5 classes	−3522.443 (40)	7289.697	0.755	0.101	183 (40.2)	170 (37.4)	50 (11)	37 (8.1)	15 (3.3)
**Men (N = 375)**									
1 class	−3172.244 (12)	6415.612	1	—	375 (100)	—	—	—	—
2 classes	−3085.932 (19)	6284.475	0.624	0.004	168 (44.8)	207 (55.2)	—	—	—
3 classes	−3039.517 26)	6233.134	0.732	0.036	105 (28.0)	222 (58.2)	48 (12.8)	—	—
4 classes	−3011.612 (33)	6218.812	0.690	0.359	84 (22.4)	166 (44.3)	67 (17.9)	58 (15.5)	—
5 classes	−2994.625 (40)	6226.327	.698	0.697	75 (20)	162 (43.2)	63 (16.8)	50 (13.3)	25 (6.7)

### Test of invariance across genders

The results of model invariance testing are reported in Table 2. In both samples, the Likelihood ratio tests indicated a statistically significant drop in model fit when parameters were fixed to be equal (constrained model M2) than when they were freely estimated (unconstrained model M1). These findings indicated a quantitative difference of latent profiles between men and women. To explore the sources of this variance, we sequentially freed the parameter of the indicator whose mean differed the most between men and women (Supplementary Table S2 reports the effect sizes of these mean differences). In Sample 1, invariance was achieved after the dimensions CARING, FEAR, and SADNESS were released from the equality constraint. In Sample 2, freeing these dimensions was not sufficient to obtain invariance, thus we also allowed PLAYFULNESS to vary across genders. Since we failed to establish equivalence for at least 3 ANPS dimensions out of 6, we considered that latent profiles were gender-specific from a quantitative point of view. Consequently, the equivalence of latent class proportions (step 3) was not tested.

**Table 2:** Models for the gender invariance of the latent profiles in Sample 1 and Sample 2. LL = Log-likelihood; −2ΔLL = Log-likelihood ratio statistic; k = number of parameters; BIC = Bayesian Information Criterion; ^1^Free to vary: SADNESS, CARING, and FEAR; ^2^Free to vary: SADNESS, CARING, FEAR, and PLAYFULNESS; ***p < 0.001; **p < 0.01; *p < 0.05.

Model *Model Comparison*	LL (k) *−2 ΔLL (Δk)*	BIC *ΔBIC*	Entropy *ΔEntropy*
**Sample 1 at T1 (N = 509)**			
M1. Unconstrained	−4364.691 (53)	9059.702	0.848
M2. Constrained	−4398.090 (35)	9014.316	0.838
*M2 vs M1*	53.620 (18)***	*−45.386*	*−0.010*
M2a Partial constrained^1^	−4373.252 (44)	9020.731	0.844
*M2a vs M1*	*11.300 (9)*	*−38.971*	*−0.004*
**Sample 2 (N = 830)**			
M1. Unconstrained	−7202.126 (53)	14760.487	0.826
M2. Constrained	−7243.164 (35)	14721.578	0.847
*M2 vs M1*	60.360 (18)***	*38.909*	*0.021*
M2a. Partial constrained²	−7206.588 (47)	14729.084	0.827
*M2a vs M1*	*6.750 (6)*	*−31.403*	*0.001*

### Description of the latent profiles

**Figure 1:**
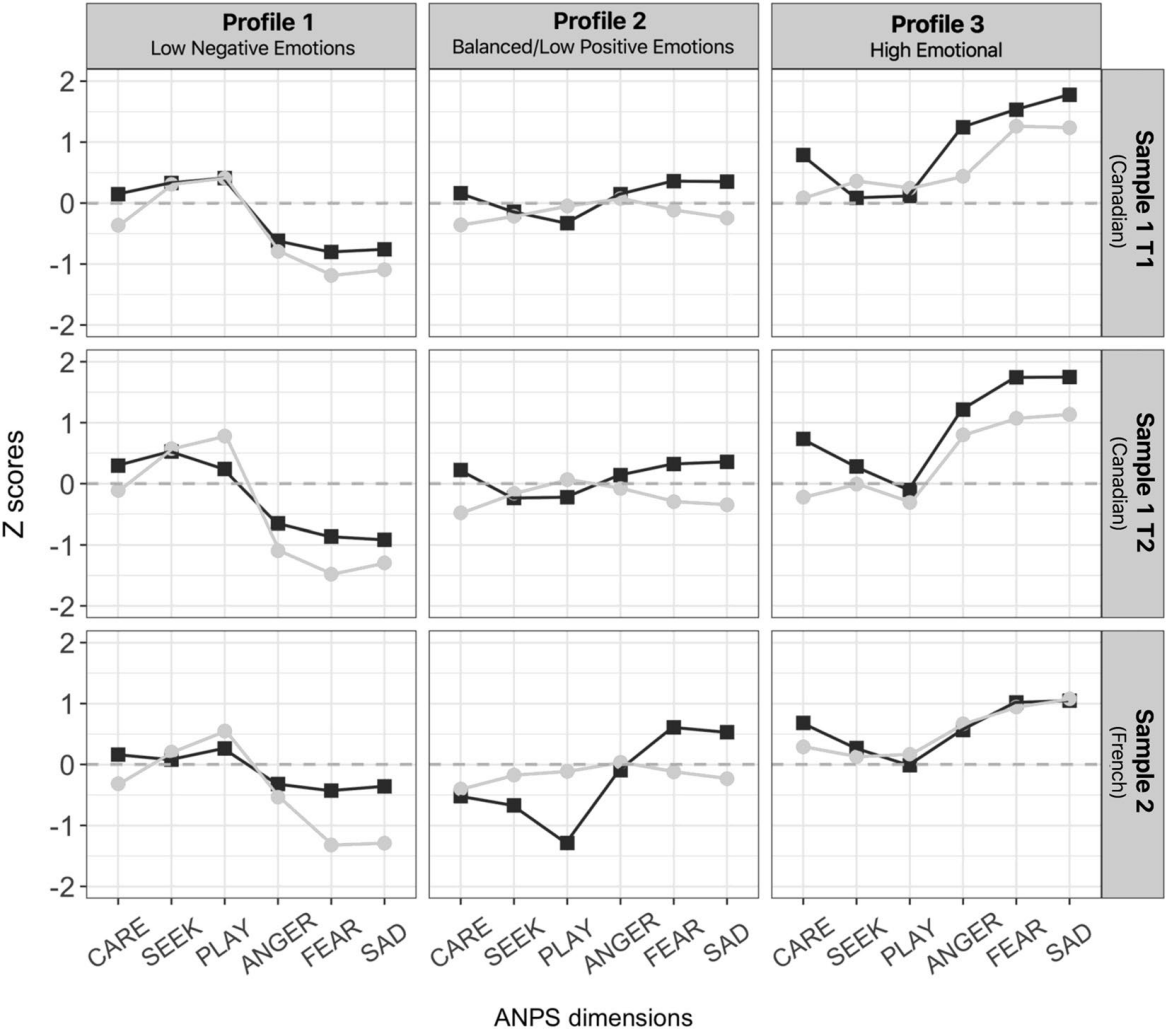
Description of the selected LPA profiles. The figure shows the profile plots for women (black lines) and men (grey lines) by sample. The scores of the 6 dimensions are the standardized estimated means (z scores).

Profiles, for men and women in both samples are presented in Fig. 1 and Supplementary Table S3. Intercorrelations among the indicators of each profile are reported in Supplementary Table S4.

In Sample 1, the profiles of men and women were qualitatively similar, with the negative ANPS dimensions that most distinguished the 3 profiles. Profile 1 had higher positive emotion scores than the overall sample mean, but the lowest values for the negative emotions compared to the other profiles (P1 = *low negative emotions*, 23.7% and 27% among women and men respectively). Profile 2, the most prevalent, had values for the positive and negative emotions that were the closest to those of the overall sample mean (P2 = *balanced profile*, 64.8% and 64% among women and men respectively). Profile 3 had the highest values of negative emotion scores and high positive emotions scores (especially the CARING/nurturing dimension) and this was even more marked among women (P3 = *high emotional*, 11.5% and 9% among women and men respectively*)*. Ethnicity did not differ across profiles for both women (*p* = 0.088) and men (*p* = 0.342). The proportion of participants having Canadian ethnical origin varied from 74% (Profile P2 = *balanced* profile) to 87% (both Profile P1 = *low negative emotions*, and Profile P3 = *high emotional*), while the proportion of participants having French ethnic origin varied from 4% (Profile P1 = *low negative emotions*) to 9% (Profile P2 = *balanced profile*) for both genders. The Supplementary Table S5 provides more details about the ethnicity among each profile and gender.

In Sample 2, Profile 1 was characterized by average positive emotion scores, and low negative emotion scores (P1 = *low negative emotions*, 49.9% and 28% among women and men respectively), as in Sample 1. Nevertheless, among women (for whom this profile was the most prevalent), the mean scores for negative emotions were closest to that of the overall sample, whereas among men these scores were lower. Profile 2 was the most prevalent among men and the shape of this profile was somewhat different across genders, with men showing a profile characterized by average scores (MP2 = *balanced profile*, 58.2%), like in Sample 1, but women (WP2 = *low positive emotions*, 14.4%) showing lower positive scores (particularly PLAYFULNESS) and higher negative ones (especially FEAR and SADNESS) compared to men. Finally, Profile 3 had the highest values of both negative and positive emotion scores, like in Sample 1 (P3 = *high emotional*, 36.0% and 12.8% among women and men respectively), and was similar across genders.

### Longitudinal stability of ANPS profiles

A prerequisite of this analysis was to verify that LPA and gender invariance analysis at T2 were consistent with our findings at T1 (Supplementary Table S6 and S7). The diagonals of the transition probability matrices presented in Table 3 showed that, in the entire Sample 1, the conditional probability of being classified at T2 in the same class as at T1 was between 88.5% and 95.4% (see also Supplementary Table S8 for the fit of the constrained versus unconstrained model). Changes in profile membership were thus very rare: (i) for the members of Profile 1 at T1 (P1 = *low negative emotions*), the conditional probability for being classified in Profile 2 at T2 was 0.8%, and in Profile 3 it was 0%; (ii) the subjects belonging to Profile 2 at T1 (P2 = *balanced profile*) had 11.5% of conditional probability for being classified in Profile 3 at T2, and 0% in Profile 3; (iii) for subjects belonging to Profile 3 (P3 = *high emotional*), the conditional probabilities for being classified in another profile were 2.6% and 2% for Profile 1 and 2 respectively. For each gender separately, these probabilities were similar, apart from Profile 2 for which the women had a 14.9% conditional probability for being classified in Profile 3 at T2 whilst it was 0% for the men.

**Table 3:** Latent Transition Probability matrices in Sample 1. The table describes the latent transition probabilities over the 4 years period in Sample 1. Each cell in the matrix represents the probability to be classified in the profile in column *j* (at T2), conditioned to the probability to have been classified in the profile in the row *i* (at T1) [P(C_j_ | C_i_)]. The diagonal element of each matrix (bold) represents no transition. The transition probability for women and men are also conditioned on gender [P(C_j_ | C_i_, Gender)].

	Profiles at T1		Profiles at T2	
Entire sample		*Profile 1*	*Profile 2*	*Profile 3*
N = 655	*Profile 1*	**0.992**	0.008	0.000
	*Profile 2*	0.000	**0.885**	0.115
	*Profile 3*	0.026	0.020	**0.954**
Women	*Profile 1*	**0.976**	0.024	0.000
N = 361	*Profile 2*	0.000	**0.851**	0.149
	*Profile 3*	0.010	0.035	**0.957**
Men	*Profile 1*	**1.000**	0.000	0.000
N = 294	*Profile 2*	0.000	**1.000**	0.000
	*Profile 3*	0.048	0.000	**0.952**

### ANPS profiles in relation to intrapersonal, interpersonal and emotion regulation variables

**Figure 2:**
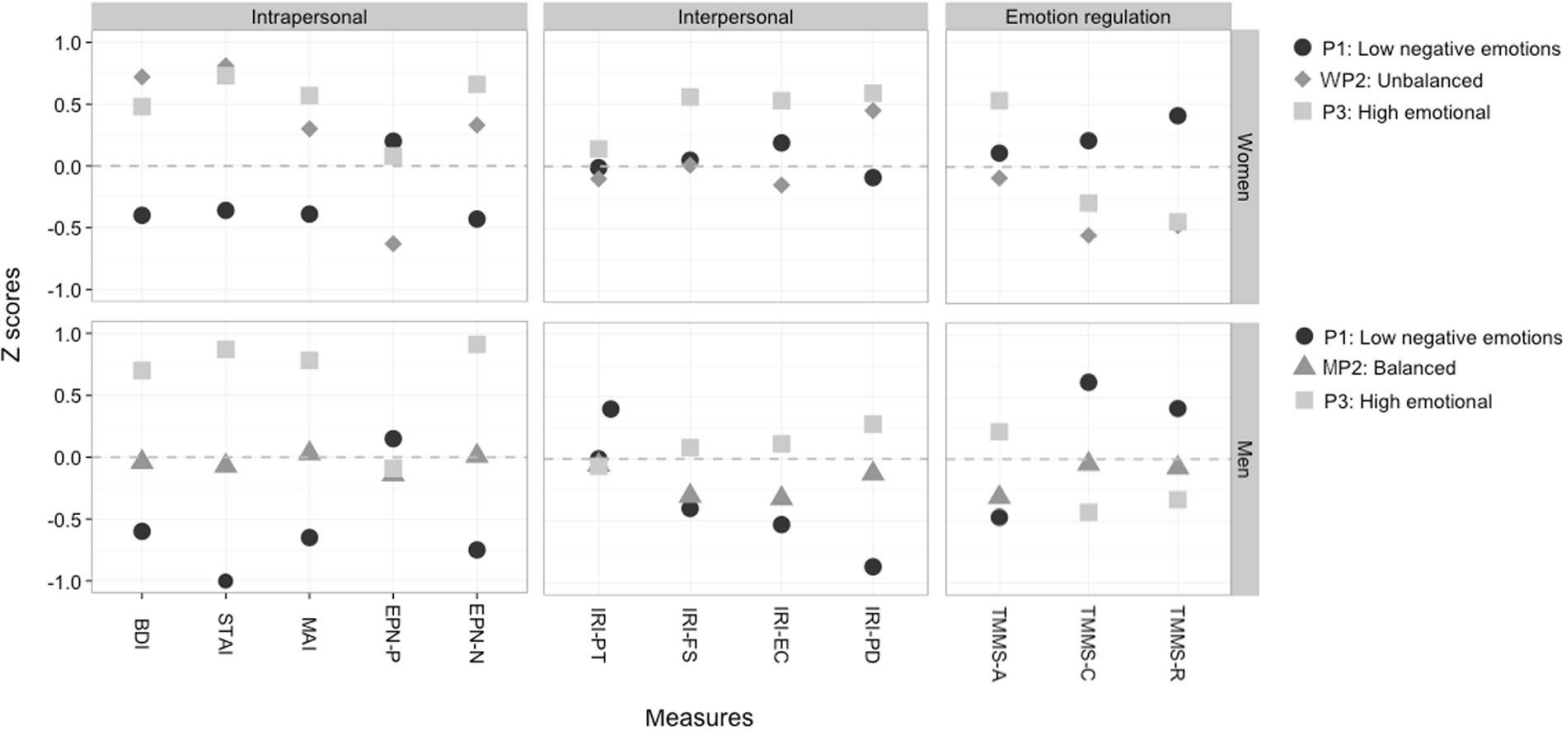
Correlates of the ANPS profiles in Sample 2. The figure represents the standardized scores (Z) of the 18 measures of intrapersonal, interpersonal and emotion regulation constructs, by profile and by gender. Profiles 1 and 3 are named the same in men and women: P1 ‘low negative emotions’, and P3 ‘high emotional’. Profile 2 is named differently in men and women (because of qualitative differences between the genders): Women WP2 ‘low positive emotions’, and Men MP2 ‘balanced’. BDI = Beck Depression Inventory short version; STAI = State-Trait Anxiety Inventory-Y; IRI = Interpersonal Reactivity Index (-PT = Perspective Taking, -FS = Fantasy Scale, -EC = Empathic Concern, -PD = Personal Distress); MAI-Multidimensional Anger Inventory; EPN-P = Positive and Negative Emotionality Questionnaire - Positive subscale; EPN-N = Positive and Negative Emotionality Questionnaire - Negative subscale; TMMS = Trait Meta Mood Scale (-A = Attention towards emotions, -C = Clarity of feelings, -R = Repair).

Table 4 and Fig. 2 report the comparisons, across the three profiles in Sample 2, of the measures of intrapersonal, interpersonal, and emotional regulation skills. For both genders the Social desirability score was significantly different across profiles, thus our statistical comparisons were adjusted for this score.

**Table 4:** ANPS profiles in relation to intrapersonal, interpersonal and emotion regulation variables in Sample 2. SD = Standard Deviation; ES = Effect Size (based on Hedge’s g); BDI-13 = Beck Depression Inventory short version; STAI-Y = Spielberger State-Trait Anxiety Inventory-Y; IRI = Interpersonal Reactivity Index; MAI = Multidimensional Anger Inventory; EPN31 = Positive and Negative Emotionality Questionnaire; TMMS = Trait Meta-Mood Scale. All p-values based on F statistic adjusted for Social Desirability. P-values for the comparisons across profiles should be interpreted as statistically significant if <0.017 due to Bonferroni correction.

	Profile 1 Low emotional		Profile 2 *Low positive emotion/Balanced*		Profile 3 *High emotional*		Comparisons			
	Mean (SD)	Min-Max	Mean (SD)	Min-Max	Mean (SD)	Min-Max	ANCOVA (p-value)	ES (p-value) 1 vs 2	ES (p-value) 1 vs 3	ES (p-value) 2 vs 3
**Women**										
**Social desirability**	5.1 (1.95)	0–9	4.17 (2.00)	0–9	4.2 (1.76)	1–8	/	0.47 (0.000)	0.48 (0.000)	−0.02 (0.905)
**Intrapersonal measures**										
BDI-13	2.02 (2.16)	0–10	6.18 (4.93)	0–21	5.29 (3.73)	0–25	0.000	−1.38 (0.000)	−1.11 (0.000)	0.22 (0.108)
STAI-Y Trait	35.79 (6.82)	20–52	47.58 (9.23)	29–76	46.82 (8.92)	23–70	0.000	−1.58 (0.000)	−1.42 (0.000)	0.08 (0.428)
MAI	98.14 (15.34)	18–135	111.03 (15.39)	64–167	116.01 (19.10)	18–162	0.000	−0.84 (0.000)	−1.05 (0.000)	−0.27 (0.032)
EPN31 - Positive	53.25 (8.51)	25–68	45.38 (10.86)	24–67	52.11 (9.04)	21–69	0.198	0.86 (0.000)	0.13 (0.238)	−0.70 (0.000)
EPN31 - Negative	43.11 (10.16)	18–74	55.03 (15.28)	28–105	60.12 (15.93)	31–115	0.000	−1.04 (0.000)	−1.32 (0.000)	−0.32 (0.027)
**Interpersonal measures**										
IRI - Perspective Taking	16.15 (3.20)	8–24	15.8 (3.28)	6–23	16.68 (3.76)	7–28	0.025	0.11 (0.899)	−0.16 (0.024)	−0.24 (0.102)
IRI - Fantasy Scale	19.43 (4.83)	7–28	19.20 (4.81)	8–28	21.95 (3.57)	12–28	0.000	0.05 (0.655)	−0.58 (0.000)	−0.69 (0.000)
IRI - Empathic Concern	19.33 (3.81)	5–27	17.83 (3.33)	9–25	20.78 (3.67)	3–28	0.000	0.40 (0.028)	−0.39 (0.000)	−0.82 (0.000)
IRI - Personal Distress	13.04 (3.87)	4–24	15.34 (3.67)	7–27	15.93 (4.09)	4–25	0.000	−0.60 (0.000)	−0.73 (0.000)	−0.15 (0.312)
**Emotional Regulation Skills**										
TMMS - Attention tw emotions	47.37 (7.85)	22–63	45.68 (8.23)	24–62	50.85 (6.19)	34–64	0.000	0.21 (0.122)	−0.48 (0.000)	−0.75 (0.000)
TMMS - Clarity of feelings	37.72 (5.95)	19–52	32.50 (6.54)	15–54	34.31 (7.20)	15–51	0.000	0.86 (0.000)	0.52 (0.000)	−0.26 (0.095)
TMMS - Repair	23.40 (3.69)	13–30	19.39 (4.54)	6–29	19.53 (5.01)	7–30	0.000	1.03 (0.000)	0.9 (0.000)	−0.03 (0.793)
**Men**										
**Social desirability**	5.43 (1.81)	1–9	4.47 (1.88)	0–9	3.60 (1.35)	1–8	/	0.51 (0.000)	1.08 (0.000)	0.48 (0.027)
**Intrapersonal measures**										
BDI-13	1.26 (1.65)	0–7	3.35 (3.47)	0–19	6.10 (5.17)	0–26	0.000	−0.69 (0.000)	−1.51 (0.000)	−0.72 (0.000)
STAI-Y Trait	28.21 (4.79)	20–44	38.76 (8.00)	21–64	48.19 (10.18)	21–77	0.000	−1.48 (0.000)	−2.87 (0.000)	−1.12 (0.000)
MAI	93.15 (17.5)	12–127	105.94 (16.94)	12–174	120.00 (14.05)	90–148	0.000	−0.75 (0.000)	−1.62 (0.000)	−0.85 (0.000)
EPN31 - Positive	52.79 (9.68)	22–68	49.96 (9.22)	24–70	50.50 (10.41)	24–69	0.016	0.30 (0.006)	0.23 (0.257)	−0.06 (0.800)
EPN31 - Negative	38.13 (11.08)	22–71	50.04 (14.22)	22–91	64.08 (15.66)	32–111	0.000	−0.89 (0.000)	−2.04 (0.000)	−0.97 (0.000)
**Interpersonal measures**										
IRI - Perspective Taking	16.16 (4.15)	6–26	16.01 (3.78)	7–24	15.96 (4.04)	5–24	0.373	0.04 (0.547)	0.05 (0.495)	0.01 (0.585)
IRI - Fantasy Scale	17.17 (5.60)	4–28	17.67 (4.87)	6–28	19.60 (4.89)	6–28	0.055	−0.10 (0.575)	−0.45 (0.056)	−0.40 (0.035)
IRI - Empathic Concern	16.18 (4.81)	5–26	17.10 (4.48)	4–27	19.02 (3.99)	10–26	0.000	−0.20 (0.004)	−0.62 (0.000)	−0.44 (0.001)
IRI - Personal Distress	9.76 (3.65)	2–20	12.92 (3.46)	4–21	14.60 (4.41)	4–24	0.000	−0.90 (0.000)	−1.23 (0.000)	−0.46 (0.005)
**Emotional Regulation Skills**										
TMMS - Attention tw emotions	42.47 (10.10)	17–65	43.86 (7.90)	22–63	48.28 (6.73)	34–63	0.000	−0.16 (0.133)	−0.63 (0.001)	−0.57 (0.000)
TMMS - Clarity of feeling	40.56 (6.30)	22–54	36.00 (6.55)	21–55	33.32 (6.81)	14–46	0.000	0.70 (0.000)	1.11 (0.000)	0.4 1(0.035)
TMMS - Repair	23.40 (3.96)	10–30	21.22 (4.07)	8–29	20.04 (4.82)	6–30	0.000	0.54 (0.000)	0.79 (0.001)	0.28 (0.195)

In both men and women, individuals in the *low negative emotions* profile (P1) reported lower score (medium to large effect size) on BDI, STAI, MAI and ENP31-negative emotion with respect to those in the other two profiles.

Regarding the interpersonal measures, medium to large differences were found between the different profiles, with larger differences for the affective dimensions of empathy (i.e., IRI Personal Distress and Empathic concern) than for the cognitive dimensions (IRI Perspective taking and Fantasy scale). In particular, regarding affective empathy men and women clustered in the *low negative emotions* profile (P1), compared to those in the *low positive emotions/balanced* (MP2/WP2) and *high emotional* profiles (P3; although the Bonferroni-corrected significance level for P1 versus P2 comparison for women was marginally significant), had lower IRI Personal Distress scores and higher IRI Empathic concern. Regarding cognitive empathy (IRI perspective taking and fantasy scores) for women, scores were higher among the *high emotional* profile (P3) than those in both the *low negative emotions* (P1) and *low positive emotions* (P2) profiles, while among men similar scores (IRI Perspective taking) were endorsed in the 3 profiles.

With respect to the emotional regulation skills, large differences were found between the different profiles, and the differences were even larger among women than among men. With respect to the TMMS Attention towards emotions, both women and men in the *high emotional* profile (P3) endorsed significantly higher scores than their counterparts in the other profiles. For the TMMS Clarity of feelings and Repair scores, and for both women and men, *low negative emotions* profile (P1) had significant higher scores than their counterparts in the two other profiles.

## Discussion

In this study, we adopted a person-centred approach to explore personality typologies among the 6 affective dimensions of the Affective Neuroscience Personality Scales. This approach has the advantage of considering personality as a dynamic system within which individual differences emerge from the associations among the basic personality traits, in line with classic theories^61^. Within Panksepp’s model of personality, these traits are linked to the functioning of 6 evolutionary-related emotional systems and operationalized through the 6 affective dimensions of the ANPS^9^. The typological bottom-up approach we used allowed us to describe, using two different samples, three profiles of personality. Despite the qualitative similarities of the profiles across genders, we found quantitative differences between men and women in the mean scores of at least three ANPS dimensions.

In the following paragraphs, we discuss in turn the gender differences, the time stability, and the relation between ANPS profiles and the other measures.

### Profile similarities and differences across samples and genders

Among young parents from Quebec (Canada), for both genders, we found a first profile characterized by low levels of negative emotions and average levels of positive emotions (P1 ‘*low negative emotions’*), a second profile characterized by average levels of emotionality (P2 ‘*balanced profile’*), and a third profile characterized by high levels of emotionality (P3 ‘*high emotional’*).

Among college students from the Paris area (France), the same three profiles identified in the Canadian sample were found for men. For women, however, the same P1 ‘*low negative emotions*’ and P3 ‘*high emotional*’ profiles were found, but there was no evidence for the P2 ‘*balanced*’ profile that was found among both Canadian men and women. Instead, French women in the second profile (WP2 ‘*low positive emotions’*) displayed an imbalanced profile, characterized by a combination of rather low levels of positive emotions (especially PLAYFULNESS) and medium levels of two core negative emotions, i.e., FEAR and SADNESS.

Beside these qualitative similarities and differences, the lack of measurement invariance across genders indicates that quantitative differences exist in the dimension means. This suggests that although the typologies among the six emotional dimensions were similar between men and women (e.g. in P1, both women and men had CARING scoring over the average and SADNESS scoring below the average; while in P3 both are over the average), these dimensions have not the same mean level in both genders (e.g. CARING is higher in women than in men). Additionally, these differences are a recurring issue in each profile: women scored higher on CARING, FEAR, and SADNESS and lower on PLAYFULNESS in each profile and sample.

As these findings are in the same direction as those of previous studies using a variable-centred approach, they ensure the validity of the latent profile classification. Indeed, the same gender differences in the mean of each indicator estimated in the LPA were found in other papers that analysed gender effects for each ANPS dimension separately^7, 34, 35, 62^. Therefore, the classification operated by the LPA did not alter the personality differences of men and women that have been described and have been shown to be consistent with the literature (i.e. greater propensity for nurturing and for anxious/depressive affect among women)^30–32^.

This being said, one discrepant finding between our two samples was observed among women classified in the second profile (Canadian: P2; French WP2). The differences between the women of the two samples in terms of country (Canada *vs*. France), age (35 *vs*. 20), socio-economic, and family status (parents *vs*. college students) may account for this discrepancy. In fact, although personality is a stable construct (according to both its definition and empirical findings, for example with the FFM), evolution in personality throughout the lifespan has also been documented^63, 64^. Most meta-analytic findings converge on considering that, at 30 years of age, personality reaches both high mean-level and rank-order stability until 70 years^65–67^, and the French women of the present study were on average 10 years younger than this age of stability. However, despite the age difference between our two samples of participants, the same profiles were found among French and Canadian men. Hence the age difference between the French and Canadian women may not fully account for the variation in the second profile. Another confounder could be the role of stressors associated with university life. Indeed, college students are considered a high-risk population for psychological difficulties^68–70^, and in a recent representative national health survey among French college students^71^, women were 2.6 fold more likely than men to manifest psychological difficulties.

### Comparison between the 2 time points

Our findings highlighted the stability of the ANPS profiles over the study period for both genders (estimated in Sample 1), as the subjects had almost perfect probability for being classified by the model as members of the same cluster over four years. Long-term predictive validity of personality types has been shown when personality was assessed with instrument relying on the FFM^72, 73^, including one study using LPTA^74^. As the ANPS is based on a different (neuro-ethological) approach, our results show for the first time evidence of the stability of personality types assessed from emotional endophenotypes. These findings are also reinforced by our previous paper showing good measurement properties of the ANPS in the context of longitudinal analysis^29^. That said, some women classified initially in the profile with average levels of emotionality (P2 ‘*balanced profile’*) appeared to have moved to the profile with high levels of emotionality (P3 ‘*high emotional’*; conditional probability: 15%) four years later, and the question whether these women are particularly sensitive to child-caring, and the potentially associated neurobiological changes, could be addressed in future studies.

### Intrapersonal, interpersonal, and emotional regulation skills

The associations between the profiles and the measured construct of intrapersonal, interpersonal, and emotion regulation skills offer evidence for the concurrent validity of the ANPS profiles.

For instance, the reported levels of anxiety, depression, anger, negative affect and interpersonal distress were lower among individuals clustered in P1 ‘*low negative emotions*’ compared to those clustered in the other profiles, with medium to very large effect sizes. We noted a clear dose-response relationship among men, with Profile 1 (P1: ‘*low negative emotions’*) having the lowest scores and Profile 3 (P3 ‘*high emotional’*) the highest, while among women the differences between the second and third profiles (respectively WP2 ‘*low positive emotions’* and P3 ‘*high emotional’)* were less marked. Further, the individuals clustered in P1 ‘*low negative emotions*’ also reported themselves to be better able to understand and regulate their emotional state (TMMS Clarity and Repair scores) than those clustered in the other profiles. These findings are consistent with the construct of emotional intelligence and the associated empirical literature on its contribution to mental health^75, 76^. They are also in line with the findings from studies using the Positive and Negative Affective Scale (PANAS), indicating that individuals with more positive emotions and fewer negative emotions tend to be more satisfied with their lives^68, 77–79^ and are positive about facing the problems and negative events of life^68, 80, 81^.

The pattern of responses of the individuals in P3 ‘*high emotional*’ provides further support for concurrent validity. These individuals reported being less able to regulate their emotions than those in the other profiles, which is consistent with elevated rumination and deficits in emotion management among those suffering from depression and/or anxiety^1, 82, 83^ (see also Borkovec’s avoidance theory of worry^84^). Furthermore, individuals in P3 reported the highest scores for most of the Interpersonal Reactivity Index subscales (except Perspective taking, for which no group differences were found), suggesting a greater propensity to imaginatively transpose themselves in the circumstances of fictional character and to experience a vicarious response to another person’s misery among people having a personality profile characterized by high levels of emotions (especially the negative ones in addition to CARING). It highlights how affectivity can influence the perception of social situations and suggests these individuals have heightened sensitivity to interpersonal situations, and easily identify with others’ emotions. Participants with this profile may also struggle to detach their own emotional states from that of others. This mechanism has been labelled ‘emotional contagion’^85, 86^, and is somewhat implied in the CARING dimension of the ANPS (e.g., item: “*I am a person who strongly feels the pain of other people”*). In line with our findings, adverse emotional reactions in response to another person’s emotional feelings (especially negative ones), empathic concern, but not poor perspective taking, are commonly reported in depressed patients^87, 88^. Our results are also coherent with the literature demonstrating that individuals who are socially anxious have elevated empathic tendencies (but similar perspective taking scores) compared to low socially anxious individuals^89^.

### Implications for future research

Findings from this study could contribute to the development of promising avenues for clinical research. Researchers interested in studying how emotional systems assessed by the ANPS emotional behaviours, cognition or health outcomes could rely on a LPA approach. This person-centred approach offers a parsimonious and holistic way of looking at personality. Indeed, studying the role of only one (or some) emotional dimension does not account for the complexity and interplay of the whole 6 affective systems described and validated by Panksepp’s model.

Replicating the present findings and extending them to clinical populations may pave the way for an adaptation of therapeutic approaches and patient management to personality profiles^90, 91^. Additionally, as the link between emotions and somatic conditions (e.g. cardiovascular diseases) is well established^92^, it could be interesting to use ANPS profiles (which are based on affective dimensions) as predictors of remission or relapse from these conditions. Findings from such studies could inform treatment strategies targeted on specific personality typologies^93^.

Developmental studies could also benefit from a person-centred approach. For instance, parental affects were shown to be associated with their offspring’s behavioural outcomes (e.g. ref. 94). However, studies rarely examined the interplay among multiple affective dimensions. Therefore, the study of the relationship between parents’ ANPS profiles and offspring’s behaviours could inform about important intergenerational effects of affective personality, and be a first step toward targeted interventions to prevent negative outcomes on children (such as intervention on parenting for at-risk families)^95^.

Besides, a long-term objective could be to address the question of the consistency of the latent profiles across a longer period (4 years could be considered a relative short time period for assessing personality changes), or even across the lifespan. For example, it could be interesting to conduct longitudinal studies focusing on the consistency of personality from childhood to adolescence and using LPTA with 3 or more time points, and compare the results with other studies that find stability of Big Five profiles^96^.

Comparisons are also needed between profiles derived from ANPS and those derived from FFM instruments. To date, the comparison between the two models has been done only from a variable-centred perspective^7, 34, 97^. Thus, examining the associations between ANPS profiles and the three personality typologies (resilients, overcontrolled, and undercontrolled) of the Big Five could substantially improve personality science, i.e. by mapping potential neurobiological bases of personality.

Finally, as the ANPS is a neurobiologically based instrument, further research is needed to address the neurobiological differences between the three latent personality profiles we described in our study.

### Limitations

The first limitation concerns the generalizability of our findings across the 2 samples. Due to the differences in the 2 populations, we cannot directly transfer the results obtained in Sample 1 to Sample 2 (and vice versa). However, the high similarity of the profiles (especially for men) supports the possibility of generalization. Nevertheless, a formal cultural comparison, over our qualitative assessment, is still required, and could be the object of future studies. Second, as LPA is sensitive to sample size, it is possible that an additional fourth profile would have emerged if our sample size were larger. Replication of our results in different populations and samples is necessary to generalize our findings across different cultures and groups. This is particularly important since LPA is an exploratory technique, and consensus about how to identify the best model is still an active research field (especially for LPTA and model invariance). Third, our two samples were not randomly selected from the population and cannot be deemed representative. Therefore, examining ANPS profiles across more varied socio-demographic populations and representative samples would be helpful to further characterize affective profiles. Fourth, the impossibility to collect data on the ethnic origin of the participants in the French sample limits our ability to assess the influence of ethnicity in the profiles emerged from our LPA. This is an important concern as French society is multicultural. Finally, the low Cronbach’s alpha of the IRI could be a concern in the evaluation of the construct examined.

## Conclusion

In conclusion, our study examined for the first time the existence of latent affective personality profiles, as measured by the Affective Neuroscience Personality Scales. We described three latent profiles in two independent samples, and evaluated their characteristics across genders, time, and in relation to external constructs. Further study should take our findings as a starting point to further corroborate the existence of these latent profiles across cultures and age groups, as well as to assess their relationships with distal outcomes.

## Electronic supplementary material

Supplementary information
