# Supplementary material for: Identifying affective personality profiles: A latent profile analysis of the Affective Neuroscience Personality Scales
Source: Sci Rep. 2017 Jul 3;7:4548. doi: 10.1038/s41598-017-04738-x (PMC5495783; doi:10.1038/s41598-017-04738-x)
Supplement: Supplementary file 1 — Supplementary information [file 41598_2017_4738_MOESM1_ESM.pdf]

Supplemental material

Identifying affective personality profiles: A latent profile analysis of the Affective Neuroscience Personality Scales.

Massimiliano Orri, Jean-Baptiste Pingault, Alexandra Rouquette, Christophe Lalanne, Bruno Falissard, Catherine Herba, Sylvana M. Côté, & Sylvie Berthoz

Contents

Table S1. Socio-demographic characteristics of the two samples 2
Table S2. Gender differences 3
Table S3. Descriptive statistics for the latent profiles in the two samples 4
Table S4. Intercorrelations among the ANPS dimensions for each profile 5
Table S5. Ethnicity for each profile by gender 6
Table S6. LPA fit statistics for the Sample 1 at T2 7
Table S7. Gender invariance assessment 8
Table S8. Longitudinal invariance of the LPA model 9
Figure S1. Latent Profile Transition Analysis model 10
References 11

Table S1. Socio-demographic characteristics of the two samples

	Sample 1	Sample 2
**Sample size**, N	509	830
**Country**	Canada	France
**Province**	Quebec	Ile-de-France
**Year of (first) data collection**	2006	2008
**Age,** mean years±SD	36.5±5.8	20.6±2.1
**Male gender,** N (%)	222 (43.6)	375 (45.2)
**Ethnicity,** N (%)		
Canadian	279 (64.6)	
French	35 (8.1)	
Other	118 (27.3)	
**Education,** N (%)		
University degree	287 (56.4)	
Partial college education	45 (8.6)	
High school grade	127 (24.7)	
Partial secondary education	40 (7.9)	
No secondary education	12 (2.4)	
**Years in college,** N (%)		
More that 2 years		259 (31.2)
Less than 2 years		571 (68.8)

Table S2. Gender differences

Men *vs.* women comparison for each profile and sample. Hedge's G (effect size) is the standardized mean difference: if negative, women have higher values than men.

The suggested interpretation of the effects size is the following[1]: <.20 = small; .21-.50 = medium; .51-.80=large; >.80=very large.

P-values are based on independent samples Student's t-tests.

	Profile 1			Profile 2			Profile 3		
	Effect size	T statistic	p-value	Effect size	T statistic	p-value	Effect size	T statistic	p-value
**Sample 1 T1**									
SEEKING	0.07	0.38	0.703	0.05	0.41	0.682	-0.22	-0.78	0.441
CARING	0.56	3.16	0.002	0.54	4.76	0.000	0.78	2.64	0.012
PLAYFULNESS	0.04	0.22	0.825	-0.31	-2.74	0.006	-0.06	-0.19	0.850
FEAR	0.66	3.79	0.000	0.73	6.74	0.000	0.41	1.42	0.164
ANGER	0.33	1.83	0.069	0.08	0.68	0.496	0.80	2.88	0.006
SADNESS	0.55	3.05	0.003	0.99	8.74	0.000	0.96	3.30	0.002
**Sample 1 T2**									
SEEKING	-0.12	-0.64	0.524	-0.07	-0.65	0.518	0.33	1.4	0.167
CARING	0.36	1.61	0.114	0.82	7.81	0.000	0.90	3.91	0.000
PLAYFULNESS	-0.58	-2.77	0.007	-0.31	-2.92	0.004	0.22	0.96	0.340
FEAR	1.14	5.75	0.000	1.00	9.64	0.000	1.12	4.84	0.000
ANGER	0.65	3.27	0.002	0.27	2.54	0.012	0.47	2.04	0.045
SADNESS	0.68	3.45	0.001	1.15	11.00	0.000	1.06	4.57	0.000
**Sample 2**									
SEEKING	0.13	-1.06	0.291	0.58	-4.97	0.000	-0.14	0.77	0.447
CARING	-0.54	4.52	0.000	0.33	-2.41	0.018	-0.42	2.54	0.013
PLAYFULNESS	0.44	-3.86	0.000	1.48	-12.43	0.000	0.25	-1.52	0.132
FEAR	-1.49	13.22	0.000	-1.21	8.18	0.000	0.02	-0.10	0.923
ANGER	-0.26	2.21	0.028	0.16	-1.21	0.229	0.24	-1.60	0.113
SADNESS	-1.52	13.11	0.000	-1.17	8.31	0.000	0.15	-0.93	0.355

Table S3. Descriptive statistics for the latent profiles in the two samples

	Profile 1			Profile 2			Profile 3		
Dimension	Mean	SD	min-max	Mean	SD	min-max	Mean	SD	min-max
**Sample 1 at T1**									
Women									
SEEKING	29.79	4.68	14-39	27.10	4.82	6-39	28.71	5.18	20-39
CARING	28.01	5.52	16-40	27.89	5.03	16-40	31.91	4.71	20-39
PLAYFULNESS	30.12	4.82	20-41	25.44	5.34	10-40	28.38	5.72	14-37
FEAR	13.35	5.08	2-25	21.86	4.96	7-33	30.91	4.47	21-40
ANGER	11.91	4.56	3-24	17.17	5.07	6-32	23.79	6.2	13-37
SADNESS	13.90	2.96	7-20	20.95	3.36	12-30	29.94	3.35	24-38
Men									
SEEKING	29.45	5.37	17-42	26.87	5.01	8-39	29.85	5.07	20-38
CARING	24.85	5.75	10-39	25.02	5.61	10-36	27.85	5.81	12-39
PLAYFULNESS	29.92	5.44	14-40	27.13	5.62	9-40	28.74	7.04	15-41
FEAR	10.33	3.91	4-21	18.54	3.92	7-28	28.90	5.28	19-39
ANGER	10.36	4.91	1-22	16.77	5.28	5-33	18.80	6.05	10-32
SADNESS	12.02	3.89	3-21	17.49	3.64	9-26	26.45	3.94	19-32
**Sample 1 at T2**									
Women									
SEEKING	30.18	4.39	19-39	26.61	4.35	9-39	29.49	5.62	19-40
CARING	28.68	5.24	14-40	28.42	4.82	17-40	31.32	4.78	22-42
PLAYFULNESS	28.15	5.05	15-40	25.51	5.48	9-41	26.26	5.36	16-37
FEAR	12.32	3.97	3-19	21.18	4.6	11-33	31.74	4.26	24-41
ANGER	11.26	4.35	1-23	16.46	4.66	5-31	23.28	6.65	7-37
SADNESS	12.85	3.31	4-21	20.4	3.4	11-30	28.43	3.28	23-36
Men									
SEEKING	30.71	3.76	24-37	26.92	4.71	11-39	27.82	4.47	20-41
CARING	26.63	6.64	14-40	24.46	4.87	8-36	26.18	6.34	13-40
PLAYFULNESS	31.20	5.51	14-42	27.12	4.99	14-39	24.94	6.15	15-40
FEAR	7.86	3.71	1-18	16.72	4.26	4-27	26.95	4.25	20-38
ANGER	8.49	4.07	2-16	15.13	5.37	3-34	20.38	5.62	9-34
SADNESS	10.66	3.00	6-21	16.46	3.44	6-30	24.89	3.33	18-32
**Sample 2**									
Women									
SEEKING	27.67	4.35	16-40	23.68	3.79	14-33	28.73	3.91	19-38
CARING	27.29	5.62	13-41	22.00	5.38	7-33	30.29	4.98	9-41
PLAYFULNESS	30.88	4.62	17-42	20.73	4.14	10-29	29.35	4.81	16-42
FEAR	19.84	4.91	5-31	27.83	4.82	18-39	30.85	4.52	19-42
ANGER	17.2	6.35	0-38	18.91	6.04	6-36	24.02	7.32	7-39
SADNESS	19.8	4.05	5-31	25.26	3.86	17-35	28.87	4.27	18-42
Men									
SEEKING	28.29	5.16	15-39	26.6	5.32	12-40	28.12	5.07	14-38
CARING	24.24	5.78	10-37	23.84	5.7	7-40	28.21	5.00	16-37
PLAYFULNESS	32.86	4.19	20-40	28.59	5.59	6-40	30.56	4.85	20-40
FEAR	12.77	4.34	1-25	22.41	4.37	9-35	30.94	5.53	20-40
ANGER	15.50	6.53	1-32	19.95	6.58	4-39	25.71	6.13	10-39
SADNESS	13.72	3.86	1-23	20.76	3.83	9-31	29.48	3.94	18-39

Table S4. Intercorrelations among the ANPS dimensions for each profile

	Sample 1						Sample 2					
	1	2	3	4	5	6	1	2	3	4	5	6
**Profile 1**												
1. SEEKING	-	.35	.17	-.08	-.06	.00	-	.15	.10	-.09	.06	-.15
2. CARING	.14	-	.36	.13	-.12	.14	.22	-	.22	.04	-.08	.30
3. PLAYFULNESS	.25	.15	-	-.09	.02	.04	.30	.29	-	.13	.16	.22
4. FEAR	-.13	-.04	-.26	-	.03	-.15	.06	.10	-.07	-	.09	.23
5. ANGER	.00	-.12	.03	.5	-	.04	-.09	.02	.10	.08	-	.03
6. SADNESS	-.01	.22	-.07	.54	.44	-	.02	.28	.06	.31	.15	-
**Profile 2**												
1. SEEKING	-	.31	.38	-.06	.04	.09	-	.23	.27	0	.07	.04
2. CARING	.33	-	.29	-.06	-.15	.14	.01	-	.27	-.06	-.21	.20
3. PLAYFULNESS	.27	.25	-	.09	-.03	.04	.02	.08	-	-.22	.07	.04
4. FEAR	-.11	.19	-.17	-	-.09	.23	-.11	.33	-.20	-	-.06	.09
5. ANGER	-.05	-.02	-.08	.38	-	-.09	-.08	.04	-.06	-.09	-	-.19
6. SADNESS	-.07	.22	-.17	.71	.45	-	-.35	.01	-.33	.48	-.21	-
**Profile 3**												
1. SEEKING	-	-.32	.52	.1	.47	-.15	-	.15	.48	.14	.08	-.34
2. CARING	.07	-	.58	-.28	-.56	.33	.04	-	.3	-.16	.01	-.06
3. PLAYFULNESS	.52	.26	-	-.14	-.15	.1	.23	.06	-	-.08	.03	-.29
4. FEAR	-.36	.11	.10	-	-.15	-.27	.10	.06	-.16	-	-.10	-.27
5. ANGER	-.11	-.43	.11	.34	-	-.17	.01	-.13	-.08	.18	-	-.12
6. SADNESS	-.49	.13	-.09	.74	.30	-	-.06	.04	-.10	.31	.10	-

For each matrix, the intercorrelations for men are reported in the upper diagonal, and the intercorrelations for women in the lower diagonal.

Table S5. Ethnicity for each profile by gender

		Ethnicity, N (%)			
		Canadian	French	Other	Missing
**Men**	Profile 1	47 (78.33)	2 (3.33)	5 (8.33)	6 (10.00)
	Profile 2	93 (65.49)	11 (7.75)	22 (15.49)	16 (11.27)
	Profile 3	14 (70.00)	1 (5.00)	1 (5.00)	4 (20.00)
**Women**	Profile 1	45 (66.18)	9 (13.24)	7 (10.29)	7 (10.29)
	Profile 2	128 (68.82)	8 (4.30)	25 (13.44)	25 (13.44)
	Profile 3	21 (63.64)	1 (3.03)	7 (21.21)	4 (12.12)

Table S6. LPA fit statistics for the Sample 1 at T2

The table reports the fit statistics for the LPA models in Samples 1 at T2. Findings are consistent with T1.

	LL (k)	BIC	Entropy	LMR (p)	Class proportion
**Sample 1 T2**					
**Women (N=319)**					
1 class	-2580.961 (12)	5231.105			
2 classes	-2497.871 (19)	5105.280	0.625	0.1872	55-45
3 classes	-2431.712 (26)	5013.319	0.806	0.0040	64-12
4 classes	-2406.268 (33)	5002.787	0.780	0.2749	55-9
5 classes	-2390.104 (40)	5010.816	0.815	0.0818	54-2
**Men (N=249)**					
1 class	-2021.404 (12)	4109.018			
2 classes	-1952.946 (19)	4010.724	0.730	0.0061	75-25
3 classes	-1912.672 (26)	3968.799	0.810	0.0511	70-14
4 classes	-1900.272 (33)	3982.621	0.832	0.4337	69-5
5 classes	-1885.995 (40)	3992.688	0.850	0.5289	67-2

LL=Log-Likelihood; k=number of estimated parameters; BIC=Bayesian Information Criteria; LMR=Vuong-Lo-Mendell-Rubin Likelihood Ratio Test (for k vs k-1 class model); Class Proportion=size (% of subjects) of the biggest and smallest class.

Table S7. Gender invariance assessment

Model<br>*Model Comparison*	Indicators<br>free to vary	LL (k)<br>*-2 ΔLL (Δk)*	Scaling<br>factor	BIC<br>*ΔBIC*	Entropy<br>*ΔEntropy*
**Sample 1 at T1 (N=509)**					
M1. Unconstrained	All	-4364.691 (53)	1.2346	9059.702	0.848
M2. Constrained	None	-4398.090 (35)	1.2288	9014.316	0.838
M2 vs M1		*53.620 (18), p=.000*		*-45.386*	*-0.010*
M2a. Partial constrained	SADNESS	-4394.971 (38)	1.1953	9026.775	0.839
M2a vs M1		*45.390 (15), p=.000*		*-32.927*	*-0.009*
M2b. Partial constrained	CARING	-4380.623 (41)	1.2208	9016.777	0.839
M2b vs M1		*24.860 (12), p=.016*		*-42.925*	*-0.009*
M2c. Partial constrained	FEAR	-4373.252 (44)	1.1774	9020.731	0.844
M2c vs M1		*11.300 (9), p=.256*		*-38.971*	*-0.004*
**Sample 1 at T2 (N=568)**					
M1. Unconstrained	All	-4733.739 (53)	1.1828	9803.610	0.882
M2. Constrained	None	-4792.878 (35)	1.1449	9807.730	0.854
M2 vs M1		*94.130 (18), p=.000*		*4.120*	*-0.028*
M2a. Partial constrained	FEAR	-4788.968 (38)	1.1273	9818.938	0.860
M2a vs M1		*83.470 (15), p=.000*		*15.328*	*-0.022*
M2b. Partial constrained	SADNESS	-4777.659 (41)	1.2482	9815.344	0.866
M2b vs M1		*91.560 (12), p=.000*		*11.734*	*-0.016*
M2c. Partial constrained	CARING	-4744.134 (44)	1.1539	9767.321	0.867
M2c vs M1		*15.700 (9), p=.073*		*-36.289*	*-0.015*
**Sample 2 (N=830)**					
M1. Unconstrained	All	-7202.126 (53)	1.1996	14760.487	0.826
M2. Constrained	None	-7243.164 (35)	1.1172	14721.578	0.847
M2 vs M1		*60.360 (18), p=.000*		*38.909*	*0.021*
M2a. Partial constrained	CARING	-7227.686 (38)	1.1163	14710.786	0.848
M2a vs M1		*36.240 (15), p=.000*		*49.701*	*0.022*
M2b. Partial constrained	SADNESS	-7223.186 (41)	1.1365	14721.950	0.845
M2b vs M1		*29.760 (12), p=.000*		*38.537*	*0.019*
M2c. Partial constrained	FEAR	-7213.216 (44)	1.2583	14722.175	0.824
M2c vs M1		*24.310 (9), p=.003*		*38.3120*	*-0.002*
M2d. Partial constrained	PLAY	-7206.588 (47)	1.1840	14729.084	0.827
M2c vs M1		*6.750 (6), p=.345*		*-31.403*	*0.001*

LL=Log-likelihood; -2ΔLL= Log-likelihood ratio statistic; k=number of parameters; BIC=Bayesian Information Criteria; Scaling factor=scaling correction factors obtained with the robust maximum likelihood estimator (MLR).

In partial invariance, the column “Indicators free to vary” represent the additional indicator that has been unconstrained, compared to the preceding model (i.e. once an indicator has been freed, it remained unconstrained in the subsequent models of the partial invariance assessment process). ***p<0.001; **p<0.01; *p<0.05

The chi-square difference test is based on loglikelihood values and scaling correction factors obtained with the MLR estimator[2].

Table S8. Longitudinal invariance of the LPA model

The table presents the comparison between an unconstrained model M1 (in which the means of the indicators are estimated freely at each time point) to a model in which the means are set to equal across time (M2). The likelihood ratio test is not significant (-2 ΔLL=9.03, k=18, p-value=0.959), indicating means invariance across time points. In addition, the constrained model also presented a better BIC value.

**Model**<br>***Model Comparison***	**LL (k)**<br>***-2 ΔLL (Δk)***	**Scaling factor**	**BIC**<br>***ΔBIC***	**Entropy**<br>***ΔEntropy***
M1. Unconstrained	-8756.874 (65)	1.1319	17935.249	0.888
M2. Constrained means	-8759.830 (47)	1.3146	17824.438	0.887
M2 vs M1	*9.030 (18), p=.959*		*-110.811*	*-0.001*

LogLik=Log-likelihood; -2ΔLL= Log-likelihood ratio statistic; k=number of parameters; BIC=Bayesian Information Criteria; Scaling factor=scaling correction factors obtained with the robust maximum likelihood estimator (MLR).

Figure S1. Latent Profile Transition Analysis model

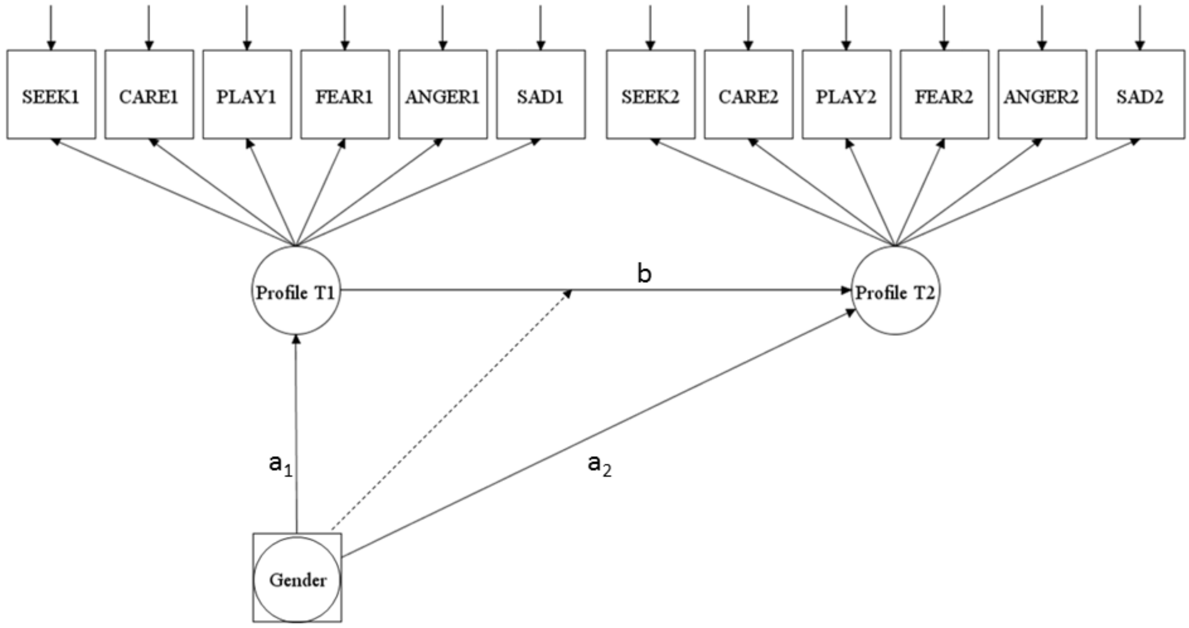

The figure represents the LTA model estimated in our study, where *a* represents the regression path between the grouping variable "gender" and the latent categorical variables "Profile" at T1 (a_1) and T2 (a_2); *b* represents the multinomial logistic regression between the latent variables "Profile T1" and "Profile T2".

References

1. Cohen, J. *Statistical Power Analysis for the Behavioral Sciences*. (Routledge, 1988).
2. Satorra, A. & Bentler, P. M. A scaled difference chi-square test statistic for moment structure analysis. *Psychometrika* **66,** 507–514 (2001).
